# Emergency Surgery and the Risk of Anastomotic Leakage After Colorectal Resection with Primary Anastomosis for Benign and Malignant Disease: A Single-Center Retrospective Study

**DOI:** 10.3390/jcm15145551

**Published:** 2026-07-15

**Authors:** Roland Sebastian Horváth, Abel Emanuel Moca, Anamaria Gozman-Pop, György Martyin, Csaba Artúr Geller, Teodor Andrei Maghiar, Octavian Adrian Maghiar, Paula Bianca Maghiar, Marius Adrian Maghiar

**Affiliations:** 1 Doctoral School of Biomedical Sciences, University of Oradea, 410087 Oradea, Romania; horvath_rolands@yahoo.com; 2 Békés County Central Hospital, 5700 Gyula, Hungary; drmartyin@gmail.com (G.M.); gellercsaba@gmail.com (C.A.G.); 3 Department of Dentistry, Faculty of Medicine and Pharmacy, University of Oradea, 410087 Oradea, Romania; 4 Bihor County Emergency Hospital, 410167 Oradea, Romania; anamaria.gozmanpop@gmail.com; 5 Department of Surgical Disciplines, Faculty of Medicine and Pharmacy, University of Oradea, 410087 Oradea, Romania; teodor.maghiar@yahoo.com (T.A.M.); octimaghiar@gmail.com (O.A.M.); pao.badea@gmail.com (P.B.M.); amaghiar@gmail.com (M.A.M.)

**Keywords:** anastomotic leakage, colorectal surgery, emergency surgery, surgical risk factors, colorectal resection, postoperative complications

## Abstract

**Background/Objectives:** Anastomotic leakage (AL) is among the most serious complications after colorectal resection with primary anastomosis. Emergency surgery has been identified as a procedural risk factor, but data from mid-volume, non-specialized centers in Central and Eastern Europe remain scarce. This study aimed to evaluate the association between emergency surgery and AL in a sample of unprotected colorectal anastomoses, while adjusting for selected clinical factors. **Methods:** We conducted a single-center retrospective observational study of consecutive adult patients undergoing left-sided or segmental colorectal resection with primary anastomosis and without protective diversion at a hospital in Gyula, Hungary, between January 2018 and December 2024, irrespective of whether the underlying indication was benign or malignant. Right colectomies, abdominoperineal resections, transanal total mesorectal excisions, Hartmann’s procedures, and diverted anastomoses were excluded. After further exclusion of patients with undocumented operative urgency, 268 patients were analyzed. AL was defined according to International Study Group of Rectal Cancer (ISREC) criteria (grade B or C). The association between emergency surgery and AL was assessed using univariable tests and an age-adjusted logistic regression model. Surgical approach was not included as a covariate because of its strong collinearity with operative urgency. **Results:** Of 268 patients, 61 (22.8%) underwent emergency surgery. AL occurred in 23 patients (8.6%). The leak rate was higher after emergency than elective surgery (19.7% vs. 5.3%; *p* = 0.001), although the confidence interval for the adjusted association was wide. Emergency surgery remained independently associated with AL after age-adjustment (adjusted odds ratio (OR) 4.35; 95% confidence interval (CI) 1.81–10.46; *p* = 0.001). No other variable was significantly associated with leakage. **Conclusions:** Emergency surgery was independently associated with an increased risk of AL in unprotected colorectal anastomoses. Operative urgency should be considered a relevant element of anastomotic risk stratification, warranting heightened vigilance and individualized protective strategies.

## 1. Introduction

AL remains the most serious complication following colorectal resection with primary anastomosis, associated with high morbidity and mortality, reintervention, prolonged hospitalization, and a negative impact on long-term survival in malignant disease [1]. Its reported incidence varies considerably across studies, ranging from approximately 2.8% to 30%, reflecting differences in the definitions applied, the location of the anastomosis, and the characteristics of the populations studied [2]. Much of this heterogeneity stems from inconsistent definitions; the International Study Group of Rectal Cancer (ISREC) proposed a consensus definition and a severity-based grading system (grades A–C) anchored on the impact on clinical management, which has since become a widely recommended framework [3]. The diagnosis is frequently established around the fifth postoperative day, although later presentations are not uncommon [4].

Risk factors for AL are classically categorized as patient-related, tumor-related, and those related to surgical technique [5]. Among procedural determinants, the elective or emergency nature of the operation has attracted particular attention. Emergency colorectal surgery represents a distinct physiologic and technical context, frequently marked by peritoneal contamination, systemic inflammation, hemodynamic instability, bowel obstruction, and impaired tissue perfusion, conditions that may individually and collectively compromise anastomotic healing [6]. Consistent with this, systematic reviews and population-based analyses have repeatedly identified emergency or urgent operation as one of the more reproducible risk factors for AL, alongside higher ASA class, open surgical approach, and advanced disease [7,8].

International benchmarking data provide useful context for interpreting institutional rates. The recent European Society of Coloproctology (ESCP) Colorectal Resection Audit (CORREA) 2022 snapshot audit reported an overall 30-day AL rate of approximately 8% and identified emergency surgery as an independent risk factor for leakage [9]. Nevertheless, observed rates are strongly influenced by local practice and patient selection, and in particular by whether protective diversion is used. The decision to fashion a defunctioning stoma is not random. It is preferentially applied to anastomoses judged to be at highest risk, and randomized evidence indicates that diversion reduces clinically relevant leak and reoperation after low anterior resection [10]. Consequently, cohorts that exclude protected anastomoses analyze a different, and potentially higher-risk, population in whom the contribution of emergency surgery to AL may be more directly observable.

Despite the large volume of data available internationally, studies specifically analyzing procedural risk factors for AL in mid-volume, non-specialized surgical centers from Central and Eastern Europe remain scarce. The demographic and practice particularities of these settings justify region-specific analyses whose results may help guide local clinical decisions [6]. The sample examined in the present study has been described previously in an analysis focused on cumulative surgeon experience and its relationship to AL [11]. That earlier report addressed surgeon- and procedure-level determinants and did not examine the elective–emergency distinction. The present study draws on the same patient population but pursues a distinct objective.

We hypothesized that emergency surgery would be associated with a higher risk of AL compared with elective surgery in this sample. Accordingly, the primary aim of the present study was to evaluate the association between emergency surgery and AL, while adjusting for selected clinical factors available in the institutional registry, in patients undergoing colorectal resection with primary anastomosis without protective diversion at a surgical center in Gyula, Hungary, over a seven-year period (2018–2024).

## 2. Materials and Methods

### 2.1. Study Design and Data Source

A single-center retrospective observational design was adopted to investigate patients treated with colorectal resection and primary anastomosis over a seven-year interval. All procedures were carried out at Békés County Central Hospital in Gyula, Hungary, and the inclusion window spanned from January 2018 through December 2024. The relevant clinical information was obtained through a structured review of institutional electronic health records, drawing on surgical protocols, hospital discharge documentation, and records of postoperative monitoring. One investigator (R.S.H.) carried out the primary data collection process over a four-month period in 2025. Because this extraction relied on a single reviewer and was not subject to duplicate coding or formal inter-rater reliability testing, a degree of systematic measurement error cannot be entirely ruled out. The sample, its eligibility framework, and the outcome definition have been reported previously [11]. Operative urgency was not part of this original 2025 extraction, and the companion analysis correctly reported this variable as unavailable at that time. For the purposes of the present study, the same investigator (R.S.H.) undertook a supplementary data-completion exercise in May 2026 to capture the elective or emergency nature of the index operation from the same institutional records. This variable remained undocumented for 14.9% of the eligible sample despite this additional effort. The present analysis therefore draws on an extended version of the original dataset, collected in two stages by the same investigator, rather than representing a partition of the same variable set analyzed previously.

### 2.2. Study Population

Eligibility was extended to all consecutive adult patients operated during the study interval who received a colorectal resection with primary anastomosis, irrespective of whether the procedure was performed in an elective or emergency setting, and whether the underlying indication was benign or malignant. Several categories of procedures were excluded:

- Right colectomies, since ileocolic anastomoses are associated with a markedly lower and more uniform leakage risk than left-sided and rectal anastomoses, and combining them with the segments of interest would have introduced unwanted heterogeneity in the outcome [7];
- Abdominoperineal resections, because no anastomosis is created;
- Transanal total mesorectal excisions, whose technical profile differs considerably from conventional approaches;
- Hartmann’s procedures;
- Cases with a diverting stoma constructed during the index operation, since fecal diversion both modifies the clinical expression of a leak and is preferentially applied to higher-risk anastomoses; their exclusion yields a sample of unprotected anastomoses in which the association between operative urgency and anastomotic leakage can be assessed more directly [12].

Application of these criteria yielded 315 patients. Because operative urgency was the primary exposure of the present study, documentation of the elective or emergency nature of the index operation in the institutional record was a further requirement for inclusion. This information was unavailable for 47 patients (14.9%), who were therefore excluded, leaving a final analytic sample of 268 patients. The same patient population, prior to the application of this additional criterion, has been described previously in an analysis addressing a different research question [11].

### 2.3. Variables

The primary outcome was anastomotic leakage (AL), treated as a dichotomous variable (present or absent). AL was defined in line with the International Study Group of Rectal Cancer (ISREC) framework, namely as a loss of integrity of the bowel wall at the anastomotic site that establishes communication between the intraluminal and extraluminal spaces [3]. Only clinically meaningful events corresponding to ISREC grade B or C, that is, leaks prompting active management such as percutaneous drainage, endoscopic intervention, or return to the operating room, were counted. A leak was registered when identified during the index admission or within 90 days of the operation, on the basis of clinical, imaging, or intraoperative evidence. For every confirmed event, the postoperative day on which the diagnosis was established was noted.

The primary exposure was the elective or emergency nature of the index operation, recorded as a dichotomous variable. Procedures were classified as emergency when performed on a non-elective, unplanned basis for an acute condition that could not be safely deferred without risk to life or organ function, in keeping with the National Confidential Enquiry into Patient Outcome and Death (NCEPOD) classification of non-elective surgery [13]. In this setting, emergency indications typically included mechanical large-bowel obstruction, perforation with peritonitis, and uncontrolled lower gastrointestinal hemorrhage. All remaining procedures, performed as planned scheduled operations, were classified as elective. The classification was based on the designation documented in the institutional operative record; patients without a documented elective or emergency designation were excluded from the analysis, as described above.

A set of additional clinical and procedural variables was assembled to characterize the sample, to permit comparison between the elective and emergency groups, and to serve as potential confounders in the multivariable analysis:

- Patient age and sex;
- Benign or malignant nature of the disease;
- Resected segment (left hemicolon, transverse colon, sigmoid, or rectosigmoid);
- Operative approach (open or laparoscopic), including any conversion to open surgery;
- Anastomotic technique (handsewn or stapled);
- Preoperative hemoglobin and serum albumin concentrations;
- Anticoagulant or antiplatelet medication;
- Systemic comorbidity status, captured both as a summary indicator (at least one documented condition vs. none) and as individual diagnoses, including arterial hypertension, ischemic heart disease, diabetes mellitus, atrial fibrillation, previous stroke, deep vein thrombosis, pulmonary embolism, myocardial infarction, and chronic venous insufficiency.

### 2.4. Statistical Analysis

Analyses were conducted in IBM SPSS Statistics, version 25 (IBM Corp., Armonk, NY, USA), with figures and tabulations prepared in Microsoft Office (Microsoft Corp., Redmond, WA, USA). The distribution of continuous measures was examined with the Shapiro–Wilk test. Depending on the result, these variables were summarized either as mean ± standard deviation or as median with interquartile range. Categorical data were reported as counts and corresponding percentages.

Two sets of comparisons were performed. First, baseline characteristics were compared between the elective and emergency groups. Second, all clinical and procedural variables were compared between patients with and without anastomotic leakage. Continuous variables that departed from normality were contrasted using the Mann–Whitney U test, while associations between categorical variables were assessed with Fisher’s exact test. A two-sided *p*-value below 0.05 was regarded as statistically significant throughout.

To evaluate the independent association between emergency surgery and anastomotic leakage, an age-adjusted binary logistic regression model was constructed, with anastomotic leakage as the dependent variable and operative urgency as the primary explanatory variable. Given the limited number of outcome events (23 leaks in the analytic sample), the number of covariates was deliberately restricted to preserve an acceptable events-per-variable ratio and limit the risk of overfitting. Age was selected a priori as an adjustment variable, on the basis of its clinical relevance and its potential to confound the association between operative urgency and leakage, and was modeled as a continuous linear term without transformation. Surgical approach was also considered but was excluded from the adjustment set because of its strong collinearity with operative urgency (open surgery in 93.4% of emergency vs. 48.8% of elective cases), which would have inflated variance without meaningfully isolating either variable’s independent contribution. Preoperative serum albumin, although significantly lower in the emergency group (38 vs. 41 g/L), was likewise not entered into the model as a covariate: because albumin plausibly lies on the causal pathway between operative urgency and leakage, reflecting the physiological consequence of urgency rather than a baseline characteristic that precedes it, adjusting for it risked biasing the total effect estimate for operative urgency rather than removing a genuine confound. Because multiple procedures in this sample were performed by the same surgeons, as previously described in a companion analysis of this population [11], the potential non-independence of observations between surgeons was considered. However, with only 23 leakage events distributed across 8 surgeons (approximately 2.9 events per cluster), the number of clusters was judged insufficient to support reliable cluster-robust or random-effects adjustment, and no such analysis was performed. Model convergence was confirmed, and no evidence of separation was observed. Calibration was assessed using the Hosmer–Lemeshow test. The conventional ten-group partition indicated poor fit (χ^2^ = 16.18, df = 8, *p* = 0.040), but several groups contained fewer than two expected events, a known source of instability with sparse outcomes; a five-group partition, recommended for models with limited outcome events, yielded a non-significant result (χ^2^ = 5.05, df = 3, *p* = 0.168). Given this sensitivity to group number, calibration is best regarded as inconclusive rather than adequately demonstrated, and this should be considered a further limitation of the adjusted model. Results of the regression were expressed as odds ratios (OR) with 95% confidence intervals (CI).

No a priori sample size calculation was performed, as the study encompassed every eligible consecutive case treated during the predefined seven-year period. Given the small number of leakage events, the available sample affords limited statistical power to detect modest associations, and the age-adjusted model, which includes only two predictors (operative urgency and age), corresponds to an events-per-variable ratio of approximately eleven, meeting the conventional minimum of ten. The findings are therefore best regarded as hypothesis-generating, requiring confirmation in larger prospective studies.

## 3. Results

### 3.1. Sample Characteristics

The final analytic sample comprised 268 patients undergoing colorectal resection with primary anastomosis and without protective diversion, for whom the elective or emergency nature of the operation was documented. Of these, 207 patients (77.2%) were operated electively and 61 (22.8%) in an emergency setting.

The sample had a male predominance (167 patients, 62.3%) and a median age of 67 years (IQR 60–74). The underlying indication was malignant in 222 patients (82.8%). The rectosigmoid was the most frequently resected segment (134 patients, 50.0%). An open approach was used in 158 procedures (59.0%) and a laparoscopic approach in 110 (41.0%). A stapled anastomosis was performed in 122 patients (45.5%). At least one systemic comorbidity was documented in 200 patients (74.6%), and anticoagulant or antiplatelet therapy in 75 (28.0%). Median preoperative haemoglobin was 129 g/L (IQR 115–142) and median serum albumin 41 g/L (IQR 37–44). The characteristics of the sample, overall and stratified by operative urgency, are summarized in Table 1.

### 3.2. Comparison of Emergency and Elective Groups

Patients operated in an emergency setting differed from elective patients in two notable respects (Table 2). Emergency procedures were performed through an open approach far more frequently than elective procedures (93.4% vs. 48.8%; *p* < 0.001). Preoperative serum albumin was significantly lower in the emergency group (median 38 vs. 41 g/L; *p* = 0.003). The two groups were otherwise comparable, with no significant differences in age (median 68 vs. 67 years; *p* = 0.530), sex (male 63.9% vs. 61.8%; *p* = 0.881), malignant pathology (86.9% vs. 81.6%; *p* = 0.440), rectosigmoid resection (50.8% vs. 49.8%; *p* = 1.000), preoperative haemoglobin (median 126 vs. 130 g/L; *p* = 0.143), or the presence of any comorbidity (82.0% vs. 72.5%; *p* = 0.180).

### 3.3. Anastomotic Leakage

Anastomotic leakage occurred in 23 of the 268 patients, corresponding to an overall incidence of 8.6%. The leak was diagnosed at a median of 5 days postoperatively (IQR 4.5–8), with most events (82.6%) presenting within the first postoperative week and a small number of late presentations extending to nine weeks. Management was predominantly operative: a Hartmann procedure was performed in 9 cases and a loop colostomy in 8, with loop transversostomy, conservative treatment, and anastomosis with loop ileostomy accounting for the remainder. One patient with anastomotic leakage died; the specific management undertaken prior to death could not be reliably reconstructed from the institutional record and is therefore reported as an outcome rather than a management category. The timing and management of leakage events are detailed in Table 3.

### 3.4. Univariable Analysis of Factors Associated with Anastomotic Leakage

On univariable analysis (Table 4), emergency surgery was strongly associated with anastomotic leakage: 12 of 61 emergency patients developed a leak (19.7%), compared with 11 of 207 elective patients (5.3%) (*p* = 0.001). None of the other variables examined reached statistical significance. Diabetes mellitus was not significantly associated with leakage (30.4% vs. 15.9%; *p* = 0.087). Age (*p* = 0.222), male sex (*p* = 0.508), malignant pathology (*p* = 0.388), rectosigmoid resection (*p* = 0.383), open approach (*p* = 0.376), stapled anastomosis (*p* = 0.282), preoperative haemoglobin (*p* = 0.595) and albumin (*p* = 0.750), anticoagulant therapy (*p* = 1.000), the presence of any comorbidity (*p* = 0.212), and arterial hypertension (*p* = 0.496) were not significantly associated with leakage.

### 3.5. Age-Adjusted Analysis and Sensitivity Analysis

Surgical approach was excluded from the adjusted model because of its strong collinearity with operative urgency (93.4% vs. 48.8% open surgery in the emergency and elective groups, respectively), which would have inflated variance without meaningfully isolating the independent contribution of either variable. In a minimally adjusted logistic regression model controlling for age only, emergency surgery remained strongly associated with anastomotic leakage (adjusted OR 4.35; 95% CI 1.81–10.46; *p* = 0.001) (Table 5). Age was not independently associated with leakage (adjusted OR 1.02 per year; 95% CI 0.97–1.07; *p* = 0.377). This estimate was virtually unchanged from the crude, univariable association (OR 4.36; 95% CI 1.82–10.48). Because operative urgency itself functions as a composite exposure reflecting indication subtype, physiologic severity, and intraoperative tissue quality that could not be measured separately, this estimate should be interpreted as the total association of emergency presentation with leakage rather than the isolated effect of operative timing alone. Given the limited event density (23 leakage events across 8 surgeons), no formal adjustment for surgeon-level clustering was undertaken; the potential contribution of surgeon-level non-independence, including differential case-mix by surgeon, to the observed association therefore cannot be excluded and is addressed further in the Limitations.

## 4. Discussion

In this single-center retrospective analysis of 268 colorectal resections with primary anastomosis and without protective diversion, emergency surgery was strongly and independently associated with AL. The leak rate was nearly four times higher after emergency than after elective operations (19.7% vs. 5.3%), and this association persisted after age-adjustment (adjusted OR 4.35; 95% CI 1.81–10.46). The minimal change in the effect estimate between the univariable and age-adjusted models indicates that the association was not materially confounded by age. The overall leakage incidence of 8.6% is consistent with contemporary international benchmarks, the recent ESCP CORREA reporting a comparable overall rate of approximately 8% [9], and is in line with the notion that, even in a mid-volume non-specialized setting, anastomotic leakage remains a frequent complication with substantial clinical and economic consequences [14].

These findings align with the broader literature identifying emergency surgery as one of the more reproducible procedural risk factors for anastomotic leakage. Large systematic reviews and meta-analyses have repeatedly reported emergency or urgent operation among the predictors of leakage [7,8], and the recent ESCP CORREA 2022 international audit identified emergency surgery as an independent risk factor, with a leak rate of 11.4% versus 7.1% in elective cases (OR 1.58) [9]. Notably, the magnitude of the association observed in the present sample was considerably larger than that reported in such benchmarking studies. One possible explanation concerns the composition of the sample: by excluding all anastomoses protected by a diverting stoma, the present analysis was restricted to unprotected anastomoses, and because diversion is preferentially applied to anastomoses judged to be at highest risk, its exclusion could remove a layer of surgical mitigation that, in unselected cohorts, attenuates the apparent contribution of emergency surgery to leakage [15,16,17]. This mechanism was not directly tested in the present study (for instance, through a sensitivity analysis comparing protected and unprotected anastomoses, or an interaction term between operative urgency and diversion status) and should therefore be regarded as a hypothesis generated by the observed effect size rather than a confirmed explanation for it. Other explanations, including differences in case-mix, surgical technique, or institutional practice relative to the benchmarking cohorts, cannot be excluded and would require direct, prospective comparison in future studies designed specifically to test this hypothesis.

The biological rationale for this association is well established. Emergency colorectal procedures are frequently performed in the context of mechanical obstruction, perforation with peritoneal contamination, systemic inflammation, and hemodynamic compromise, all of which impair local tissue perfusion and the healing capacity of the anastomosis [6,18]. The absence of mechanical bowel preparation and the often suboptimal physiological status of patients requiring urgent operation further contribute to this risk [6]. Consistent with this, preoperative serum albumin in the present sample was significantly lower in the emergency group (median 38 vs. 41 g/L; *p* = 0.003), reflecting the poorer nutritional and inflammatory status that characterizes emergency presentations. However, albumin itself was not significantly associated with leakage in this sample (41 g/L in patients with and without AL; *p* = 0.750), so while a mediating role for albumin between operative urgency and leakage remains biologically plausible, it should be regarded as an untested hypothesis rather than a relationship demonstrated by the present data.

Surgical approach was not included in the adjusted model because of its strong collinearity with operative urgency: open surgery was used in the large majority of emergency procedures (93.4%), compared with fewer than half of elective procedures, reflecting the reality that urgent operations are rarely amenable to a laparoscopic approach. Had the approach been retained as a covariate, the resulting estimate would have been difficult to interpret, a difficulty well recognized in the literature. In a nationwide American College of Surgeons National Surgical Quality Improvement Program (ACS-NSQIP) analysis of more than 23,000 elective colorectal resections, the laparoscopic approach was associated with lower adjusted odds of anastomotic leakage, but the authors emphasized that laparoscopic patients were systematically younger and had fewer comorbidities, reflecting substantial selection differences between approaches [19]. Similarly, a large claims-based analysis found reduced leakage odds with laparoscopy but cautioned that patient selection and conversion to open surgery are major confounders that retrospective designs cannot fully disentangle [20]. In the present sample, where the laparoscopic approach was largely confined to elective operations, these considerations are magnified, and surgical approach is best regarded as a marker of operative context rather than an independently estimable determinant of leakage in this dataset.

Among the comorbidities examined, diabetes mellitus was not significantly associated with leakage (30.4% vs. 15.9%; *p* = 0.087). Given the small number of events, this finding does not exclude a possible association. The observed numerical difference is directionally consistent with the established role of diabetes in impaired anastomotic healing, which is thought to operate through diabetic microvascular injury, including hyaline degeneration of small arteries, basement membrane thickening, and luminal narrowing that compromise local microcirculation and tissue perfusion at the anastomotic site [18]. Nonetheless, the limited number of events precludes any firm conclusion regarding diabetes in this sample.

From a clinical standpoint, and with the hypothesis-generating nature of this study in mind, these findings are consistent with treating operative urgency as one relevant element of risk stratification at the time of surgery. In the emergency setting, where the leak rate approached one in five, this risk should prompt consideration of the full range of intraoperative and perioperative mitigation strategies available for an unprotected, high-risk anastomosis, rather than diversion alone. These include intraoperative perfusion assessment, for which randomized evidence remains mixed, individual RCTs have generally been neutral, while meta-analyses of pooled RCT data suggest a benefit, particularly for left-sided resections [21]; anastomotic reinforcement techniques, whose effectiveness across multiple approaches was recently synthesized in an umbrella review of meta-analyses [22]; and a defunctioning stoma with a planned early closure interval, rather than an indefinite one. The present study cannot adjudicate among these strategies, since none of the relevant intraoperative parameters (perfusion assessment, reinforcement technique, or stoma decision-making) were recorded. The appropriate balance among them, in the specific context of emergency, unprotected anastomoses, therefore remains an open question for prospective study. Closer postoperative surveillance and early recognition of leakage remain warranted regardless of the intraoperative strategy chosen, particularly in patients with additional adverse features such as hypoalbuminemia. The relevance of nutritional status in this context is well supported: in a cohort of 170 patients undergoing emergency colorectal surgery, Pishgahi et al. found that lower preoperative serum albumin was significantly associated with the development of postoperative complications, including anastomotic leakage and surgical site infection, and proposed albumin as a practical tool for identifying higher-risk patients in precisely the setting where preoperative optimization is most constrained [23]. This is consistent with the lower albumin observed in our own emergency group and with broader evidence that hypoalbuminemia, even when modest, adversely influences healing and postoperative outcomes after colorectal surgery [24]. At the same time, the predictive value of albumin should not be overstated; in an elective Enhanced Recovery After Surgery (ERAS) cohort, Choi et al. reported that preoperative albumin had limited positive predictive value for anastomotic leakage when assessed in isolation, underscoring that it is best interpreted as one component of a broader risk profile rather than a standalone marker [25]. While such measures must be individualized, the substantial difference in leak rates between emergency and elective operations suggests that the circumstances of the operation, and not only patient- or technique-related factors, may materially influence anastomotic outcomes, a hypothesis that warrants confirmation in larger prospective studies.

Surgical site infection (SSI) constitutes the most frequent postoperative complication following colorectal surgery, and beyond the associated pain and prolonged wound care, it is linked to longer hospitalization, hospital readmission, increased healthcare costs, and, in more severe presentations, progression to sepsis and death [26]. Among abdominal procedures, colorectal surgery carries the highest reported SSI rates, cited between 15% and 30% depending on surveillance methodology and case mix [27]. Gram-negative enteric organisms predominate among isolated pathogens, with *Escherichia coli* most frequently identified, followed by *Klebsiella pneumoniae*, *Pseudomonas aeruginosa*, and *Acinetobacter* species, consistent with translocation of colonic flora into the operative field [28]. This progression from localized infection to systemic sepsis has been examined directly in prospective colorectal surgery cohorts: in a prospective single-center study of 141 patients undergoing elective colorectal cancer resection, postoperative sepsis developed in 12.8% of cases, with anastomotic leakage identified as its most frequent underlying cause, and age over 65 years, American Society of Anesthesiologists (ASA) class >2, diabetes, and cardiovascular comorbidity emerging as predisposing factors [29]. These findings reinforce that anastomotic leakage, the focus of the present study, is not only a clinically significant endpoint in itself but also a proximate driver of postoperative sepsis, further underscoring the relevance of risk stratification in the emergency, unprotected-anastomosis setting examined here. Although the present study did not systematically analyze SSI as an outcome (see Limitations), several of its established risk factors, including open surgical approach, hypoalbuminemia, and emergency presentation, overlap with those examined here for anastomotic leakage, suggesting that the elevated risk observed in the emergency group in this cohort may extend beyond anastomotic integrity to wound-related morbidity more broadly.

Beyond established inflammatory markers such as C-reactive protein, attention has recently turned to novel biomarkers reflecting the systemic inflammatory response to surgical stress. Among these, butyrylcholinesterase (BChE), a non-specific cholinesterase enzyme whose serum levels decline during systemic inflammation and sepsis, has emerged as a promising candidate for predicting infectious complications after colorectal surgery. In a prospective single-center study of 402 patients, lower BChE levels on the first and third postoperative days were independently associated with a more than two-fold higher risk of developing surgical site infection, with significantly lower levels also observed among affected patients through the fifth postoperative day [30]. While the present study did not measure BChE or other novel biomarkers, its incorporation into future risk-stratification protocols for emergency, unprotected colorectal anastomoses represents a promising direction for prospective validation.

Several limitations should be acknowledged, and together they argue for a cautious, hypothesis-generating interpretation of the findings rather than a confirmatory one. The retrospective single-center design limits the extent to which the findings can be applied to other settings and exposes the analysis to information and selection bias inherent to this type of study. The study was designed around anastomotic leakage as the sole outcome of interest; other postoperative complications, such as surgical site infection, sepsis, or intra-abdominal abscess, were not systematically analyzed and are not reported here. The number of leakage events was small (23 in the analytic sample), which constrains the reliability of the multivariable analysis. Although the logistic regression model was deliberately restricted to two predictors (operative urgency and age) to preserve an acceptable events-per-variable ratio, and this ratio (approximately eleven) now meets the conventional minimum of ten, the small absolute number of events still limits precision, and the adjusted estimates should be interpreted with appropriate caution. Operative urgency was not documented for 47 patients (14.9%), who were excluded from the analysis. These patients had a leakage rate (4.3%) comparable to that of the elective group, making systematic bias from their exclusion unlikely, although it cannot be entirely excluded. Several established determinants of anastomotic leakage risk could not be incorporated into the model, including ASA classification, body mass index, smoking status, anastomotic height from the anal verge, the presence of obstruction or perforation as distinct entities, intraoperative blood loss, detailed nutritional status beyond serum albumin and cumulative surgeon experience, which was examined in a companion analysis of this same cohort but was not entered as a covariate here in order to keep the model parsimonious given the limited number of events [11,31]. Operative urgency also functioned as a composite exposure, aggregating indication subtype (obstruction, perforation, or hemorrhage), physiologic severity (no shock index, lactate, or ASA score available), and intraoperative tissue quality (perfusion and bowel-wall integrity not assessed). The adjusted odds ratio therefore reflects this composite construct rather than operative timing alone. Among these, ASA classification, body mass index, and immunosuppressive status represent genuine baseline confounders that could have been adjusted for had they been available; by contrast, acute physiologic severity and intraoperative tissue quality are better conceptualized as mediators on the causal pathway between operative urgency and leakage, and their absence from the model is therefore not a source of confounding bias but a limitation on the ability to decompose the total effect into its underlying mechanisms. Because these variables could not be accounted for, residual confounding cannot be excluded, and the adjusted association between emergency surgery and leakage should be interpreted with this constraint in mind. The strong correlation between surgical approach and operative urgency in this sample (open surgery in 93.4% of emergency versus 48.8% of elective cases) was the basis for excluding surgical approach from the adjusted model, since its inclusion would have inflated variance without meaningfully isolating either variable’s independent contribution. As a consequence, however, the reported estimate for emergency surgery should be understood as reflecting a composite of operative urgency and its close correlate, surgical approach, rather than the fully isolated effect of operative timing alone. The small number of events per surgeon (23 events across 8 surgeons) precluded any reliable adjustment for surgeon-level clustering, whether through cluster-robust standard errors or a formal random-effects model. Residual surgeon-level confounding, including differential case-mix across surgeons, therefore cannot be excluded and should be regarded as an unaddressed source of uncertainty in the adjusted estimate. The classification of urgency relied on the designation recorded in the operative documentation rather than on a prospectively applied protocol. Ascertainment of leakage may not have been uniform across groups, since emergency patients typically receive closer monitoring and more frequent imaging, which could inflate apparent incidence independent of true risk; this is only partly offset by restricting the outcome to ISREC grade B/C events. Finally, the sample derives from the same institutional population previously reported in an analysis of cumulative surgeon experience [11]. The present study addresses a distinct research question and analytic sample, but shares the underlying data source. Taken together, these constraints mean that the present findings are best regarded as hypothesis-generating rather than definitive, and they require confirmation in larger, prospective, multicenter cohorts with more comprehensive risk adjustment.

## 5. Conclusions

In this retrospective analysis, emergency surgery was independently associated with an increased risk of anastomotic leakage, with a leak rate approaching one in five emergency cases compared with approximately one in twenty elective cases. The association persisted after age-adjustment and was larger than that reported in unselected benchmarking cohorts, consistent with the exclusion of diversion-protected anastomoses. Given the retrospective single-center design, the small number of leakage events, and the restricted set of covariates available for adjustment, these findings should be regarded as hypothesis-generating rather than definitive. With this caveat, they support the consideration of operative urgency as a relevant factor in anastomotic risk stratification and suggest a need for heightened vigilance and individualized protective strategies in the emergency setting. Larger prospective multicenter studies with comprehensive risk adjustment are needed to confirm these observations.

## Figures and Tables

**Table 1:** Characteristics of the study sample.

Characteristic	Value
Operative urgency, *n* (%)	
Elective	207 (77.2)
Emergency	61 (22.8)
Age (years)	
Median (IQR)	67 (60–74)
Sex, *n* (%)	
Male	167 (62.3)
Female	101 (37.7)
Pathology, *n* (%)	
Malignant	222 (82.8)
Benign	46 (17.2)
Resected segment, *n* (%)	
Rectosigmoid	134 (50.0)
Sigmoid	73 (27.2)
Left hemicolon	29 (10.8)
Transverse	32 (11.9)
Surgical approach, *n* (%)	
Open	158 (59.0)
Laparoscopic	110 (41.0)
Anastomotic technique, *n* (%)	
Stapled	122 (45.5)
Handsewn	146 (54.5)
Comorbidities, *n* (%)	
Any comorbidity	200 (74.6)
No comorbidity	68 (25.4)
Anticoagulant therapy, *n* (%)	
Present	75 (28.0)
Absent	193 (72.0)
Preoperative laboratory, median (IQR)	
Hemoglobin (g/L)	129 (115–142)
Serum albumin (g/L)	41 (37–44)

**Table 2:** Comparison of baseline characteristics between elective and emergency groups.

Characteristic	Elective (*n* = 207)	Emergency (*n* = 61)	*p* *
Age (years), median (IQR)	67 (61–74)	68 (59–75)	0.530
Male sex, *n* (%)	128 (61.8)	39 (63.9)	0.881
Malignant pathology, *n* (%)	169 (81.6)	53 (86.9)	0.440
Rectosigmoid resection, *n* (%)	103 (49.8)	31 (50.8)	1.000
Open approach, *n* (%)	101 (48.8)	57 (93.4)	<0.001
Stapled anastomosis, *n* (%)	96 (46.4)	26 (42.6)	0.664
Any comorbidity, *n* (%)	150 (72.5)	50 (82.0)	0.180
Anticoagulant therapy, *n* (%)	58 (28.0)	17 (27.9)	1.000
Preoperative hemoglobin (g/L), median (IQR)	130 (116–142)	126 (110–139)	0.143
Preoperative albumin (g/L), median (IQR)	41 (37–44)	38 (34–42)	0.003

* Mann–Whitney U test (continuous); Fisher’s exact test (categorical).

**Table 3:** Timing and management of anastomotic leakage events.

Feature	Value
Onset, median (IQR), days	5 (4.5–8)
Onset range, days	3–63
Early presentation (≤POD 7), *n* (%)	19 (82.6)
Late presentation (>POD 7), *n* (%)	4 (17.4)
Management, *n* (%)	
Hartmann procedure	9 (39.1)
Loop colostomy	8 (34.8)
Loop transversostomy	2 (8.7)
Conservative treatment	2 (8.7)
Anastomosis with loop ileostomy	1 (4.3)
Not determinable (patient died before management could be recorded)	1 (4.3)

**Table 4:** Univariable comparison of clinical and procedural variables by anastomotic leakage status.

Variable	No AL (*n* = 245)	AL (*n* = 23)	*p* *
Emergency surgery, *n* (%)	49 (20.0)	12 (52.2)	0.001
Age (years), median (IQR)	68 (62–73)	69 (66–76)	0.222
Male sex, *n* (%)	151 (61.6)	16 (69.6)	0.508
Malignant pathology, *n* (%)	201 (82.0)	21 (91.3)	0.388
Rectosigmoid resection, *n* (%)	120 (49.0)	14 (60.9)	0.383
Open approach, *n* (%)	142 (58.0)	16 (69.6)	0.376
Stapled anastomosis, *n* (%)	109 (44.5)	13 (56.5)	0.282
Preoperative hemoglobin (g/L), median (IQR)	129 (116–143)	127 (116–141)	0.595
Preoperative albumin (g/L), median (IQR)	41 (36–44)	41 (37–42)	0.750
Anticoagulant therapy, *n* (%)	69 (28.2)	6 (26.1)	1.000
Any comorbidity, *n* (%)	180 (73.5)	20 (87.0)	0.212
Arterial hypertension, *n* (%)	159 (64.9)	13 (56.5)	0.496
Diabetes mellitus, *n* (%)	39 (15.9)	7 (30.4)	0.087

* Fisher’s exact test (categorical variables); Mann–Whitney U test (continuous variables).

**Table 5:** Univariable and minimally adjusted logistic regression for the association between emergency surgery and anastomotic leakage.

Variable	AL, *n*/*N* (%)	Univariable OR (95% CI)	*p*	Adjusted OR (95% CI)	*p*
Operative urgency					
Elective	11/207 (5.3)	1 (reference)	—	1 (reference)	—
Emergency	12/61 (19.7)	4.36 (1.82–10.48)	0.001	4.35 (1.81–10.46)	0.001
Age (per year)	—	1.02 (0.97–1.07)	0.351	1.02 (0.97–1.07)	0.377

## Data Availability

The data presented in this study are not publicly available due to privacy and ethical restrictions. The data are held by Békés County Central Hospital, Gyula, Hungary, and may be made available upon reasonable request to the corresponding author, subject to institutional approval.
